# Supplementary material for: Sperm rDNA Copy Number and Methylation Are Associated with Male-Factor Infertility
Source: Int J Mol Sci. 2025 Nov 1;26(21):10657. doi: 10.3390/ijms262110657 (PMC12609593; doi:10.3390/ijms262110657)
Supplement: Supplementary file 1 [file ijms-26-10657-s001.zip › ijms-3919462-supplementary/Supplementary Tables S2-S4.pdf]

Table S2. Comparison of promoter methylation, absolute and presumably active CN between NSP ($N = 94$) and different ASP subgroups.

**ASP Subgroup**	***N***	***p* Value for a Between-Group Difference in** **Methylation**	**Absolute CN**	**PresumablyActive CN**
Oligoasthenoteratozoospermia (OAT)	37	0.023	0.923	0.058
Asthenozoospermia *	49	0.002	0.468	0.032
Oligozoospermia *	6	0.079	0.212	0.057
Cryptozoospermia *	5	0.089	0.415	0.066
Teratozoospermia *	13	0.017	0.415	0.020

* not including OAT samples.

Table S3. Primers for ddPCR of human rDNA.

**Assay**	**Primer**	**Sequence (5'-3')**
28S rDNA	Forward	5’-AACGTGAGCTGGGTTTAG-3’
	Reverse	5’-CTCGTACTGAGCAGGATTAC-3’
	Probe	5’-/5HEX/TGGCAACAA/ZEN/CACATCATCAGT/3IABkFQ/-3’
TBP	Forward	5’-GATATGAGACTGTGGGTAAGT-3’
	Reverse	5’-GATCCTTTGAACACCCTAATG-3’
	Probe	5’-/56-FAM/ACAGAGATC/ZEN/ACTGCAGTTGC/3IABkFQ/-3’

Table S4. Primers for deep bisulfite sequencing of the human rDNA promoter (UCE/CP) region.

**Primer**	**Sequence (5'-3')**	**Amplicon Length**	**Variant**	**Annealing Temp. (°C)**	**No. of CpGs**
Forward	TATTYGGAGGTTTAATTTTTTTAG	239 bp	A/G	56°C	25
Reverse	TATATCCTAAAATTAACCAAAAAACCCC				
